# Medical Education and Artificial Intelligence: Web of Science–Based Bibliometric Analysis (2013-2022)

**DOI:** 10.2196/51411

**Published:** 2024-10-10

**Authors:** Shuang Wang, Liuying Yang, Min Li, Xinghe Zhang, Xiantao Tai

**Affiliations:** 1 Second Clinical Medical College, Yunnan University of Chinese Medicine, Kunming, China

**Keywords:** artificial intelligence, medical education, bibliometric analysis, CiteSpace, VOSviewer

## Abstract

**Background:**

Incremental advancements in artificial intelligence (AI) technology have facilitated its integration into various disciplines. In particular, the infusion of AI into medical education has emerged as a significant trend, with noteworthy research findings. Consequently, a comprehensive review and analysis of the current research landscape of AI in medical education is warranted.

**Objective:**

This study aims to conduct a bibliometric analysis of pertinent papers, spanning the years 2013‐2022, using CiteSpace and VOSviewer. The study visually represents the existing research status and trends of AI in medical education.

**Methods:**

Articles related to AI and medical education, published between 2013 and 2022, were systematically searched in the Web of Science core database. Two reviewers scrutinized the initially retrieved papers, based on their titles and abstracts, to eliminate papers unrelated to the topic. The selected papers were then analyzed and visualized for country, institution, author, reference, and keywords using CiteSpace and VOSviewer.

**Results:**

A total of 195 papers pertaining to AI in medical education were identified from 2013 to 2022. The annual publications demonstrated an increasing trend over time. The United States emerged as the most active country in this research arena, and Harvard Medical School and the University of Toronto were the most active institutions. Prolific authors in this field included Vincent Bissonnette, Charlotte Blacketer, Rolando F Del Maestro, Nicole Ledows, Nykan Mirchi, Alexander Winkler-Schwartz, and Recai Yilamaz. The paper with the highest citation was “Medical Students’ Attitude Towards Artificial Intelligence: A Multicentre Survey.” Keyword analysis revealed that “radiology,” “medical physics,” “ehealth,” “surgery,” and “specialty” were the primary focus, whereas “big data” and “management” emerged as research frontiers.

**Conclusions:**

The study underscores the promising potential of AI in medical education research. Current research directions encompass radiology, medical information management, and other aspects. Technological progress is expected to broaden these directions further. There is an urgent need to bolster interregional collaboration and enhance research quality. These findings offer valuable insights for researchers to identify perspectives and guide future research directions.

## Introduction

The concept of artificial intelligence (AI), referring to machines and systems capable of emulating human intelligence, was first introduced at an academic conference in 1956. Its extensive research fields encompass numerous domains, including intelligent expert systems, language processing, intelligent data retrieval, and intelligent control. AI stands as one of the three groundbreaking technologies of the 21st century, sharing the pedestal with genetic engineering and nanoscience technologies [1-3]. The ultimate aim of AI is to facilitate the use of machines in replicating and expanding human intelligence. In doing so, machines are empowered to listen, see, speak, think, and make decisions in a manner akin to humans, thus elevating the quality of human life [4,5].

The sustained evolution of AI has resulted in a paradigm shift in medical practice, transitioning from traditional methods to digital health care, with AI finding applications in diverse realms of medical and health care. AI can generate pathological diagnostic reports through integrated data analysis, aid psychologists in diagnosing mental disorders by simulating human thinking patterns, and perform imaging evaluations via deep learning. Moreover, AI can be used to manage clinical patients, and deliver doctor-prescribed treatment plans through records of patient history and treatment processes [6]. Research in AI has demonstrated that the output-input ratio in the medical field holds more promise than other disciplines [7]. As such, the advancement of medical education is imperative, and, over the past several decades, research and development in the application of AI in medical education has escalated [8].

Bibliometrics serves as a tool for the quantitative analysis of published literature, determining the relationship between research statements and emerging research frontiers, based on co-occurrence, citation, and cocitation [9]. Numerous global bibliometric analyses have been conducted using CiteSpace and VOSviewer in recent years. These analyses have focused on the comprehensive rehabilitation statuses and research trends of diseases such as cancer, ankylosing spondylitis, motor and neuropathic pain, and osteoarthritis [10-13]. However, to the best of our knowledge, a bibliometric analysis of AI’s application in medical education has yet to be implemented.

Consequently, this study leverages CiteSpace and VOSviewer to assess the current research status and emergent trends of AI in medical education over the past decade.

## Methods

All data for this research were procured from the Web of Science. The search parameters for data retrieval encompassed the topics “artificial intelligence” and “medical education” (refer to Table 1), with a publication date range from 2013 to 2022. The search results were subsequently analyzed using CiteSpace and VOSviewer. CiteSpace, a visual analysis software developed by Chaomei Chen, was used to analyze the total number of papers related to the topic, the trend of changes over the years, the frequency of keywords, and centrality. This software allowed for a more convenient and intuitive analysis of the structure, rules, and distribution of subject knowledge. A scientific knowledge map facilitated the identification of research hotspots, progress, and the current situation within a specific field. VOSviewer, a software tool primarily oriented toward document data processing, enabled the analysis of the country, institution, author, journal, keywords, and co-occurrence knowledge graph of country, institution, journal, and document in the literature. Each node on the knowledge graph represented a unique element, with the connection width between nodes indicating collaboration strength, node size reflecting the number of publications, and larger nodes indicating more frequent releases.

**Table 1.** Search queries.

Set	Results, n	Search query
#1	140,447	(((TS^a^=(generative AI))^b^ OR TS=(AI)) OR TS=(Artificial Intelligence)) OR TS=(generative Artificial Intelligence)Indexes=Web of Science, timespan=2013-2022
#2	93,678	(TS=(medical education)Indexes=Web of Science, timespan=2013-2022
#3	580	#1 and #2

a TS: topic.

b AI: artificial intelligence.

The papers for this study were downloaded in .txt format from the Web of Science database. Two expert researchers examined the title, keywords, and abstract, and screened the papers based on inclusion and exclusion criteria. In cases of disagreement or difficulty in paper inclusion, a third reviewer made the final decision via discussion. Initially, a total of 580 papers were searched, of which 385 papers that did not meet the study’s topic were excluded, resulting in the retention of 195 papers.

### Ethical Considerations

According to the Regulations of the People’s Republic of China on Ethical Review of Science and Technology (Trial), Number 167 of the State Science and Technology Development Supervision (2023), scientific research activities involving humans or other animals need to undergo ethical review. This thesis does not involve humans or other animals, nor does it pose risks to life and health, the ecological environment, public order, or sustainable development. Therefore, ethical approval is not required.

## Results

### Annual Publications

**Figure 1.**
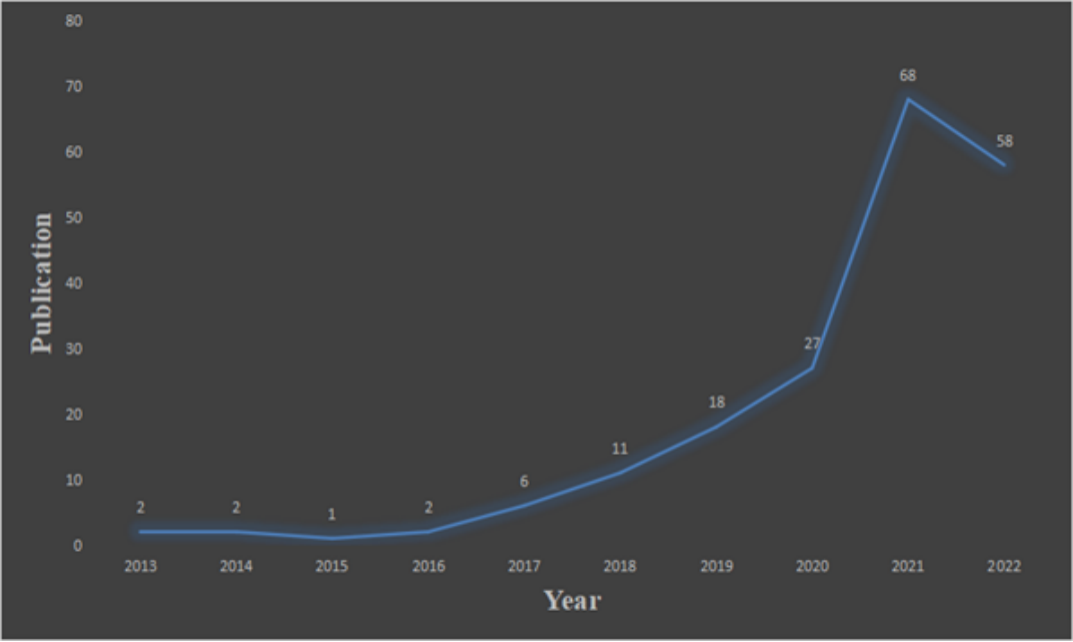
Chart of the number of years issued.

Figure 1 shows that a total of 195 papers on AI and medical education have been published in the past decade, showing an overall upward trend. The publications saw a significant surge from 2020 to 2021, reaching a peak in 2021, although the number of related papers published in 2022 decreased. The development of AI presented unprecedented opportunities and challenges to the medical and health industry. Medical education, being the cornerstone of medical industry development, can benefit from the application of AI, driving continual innovation.

### National Analysis

**Figure 2.**
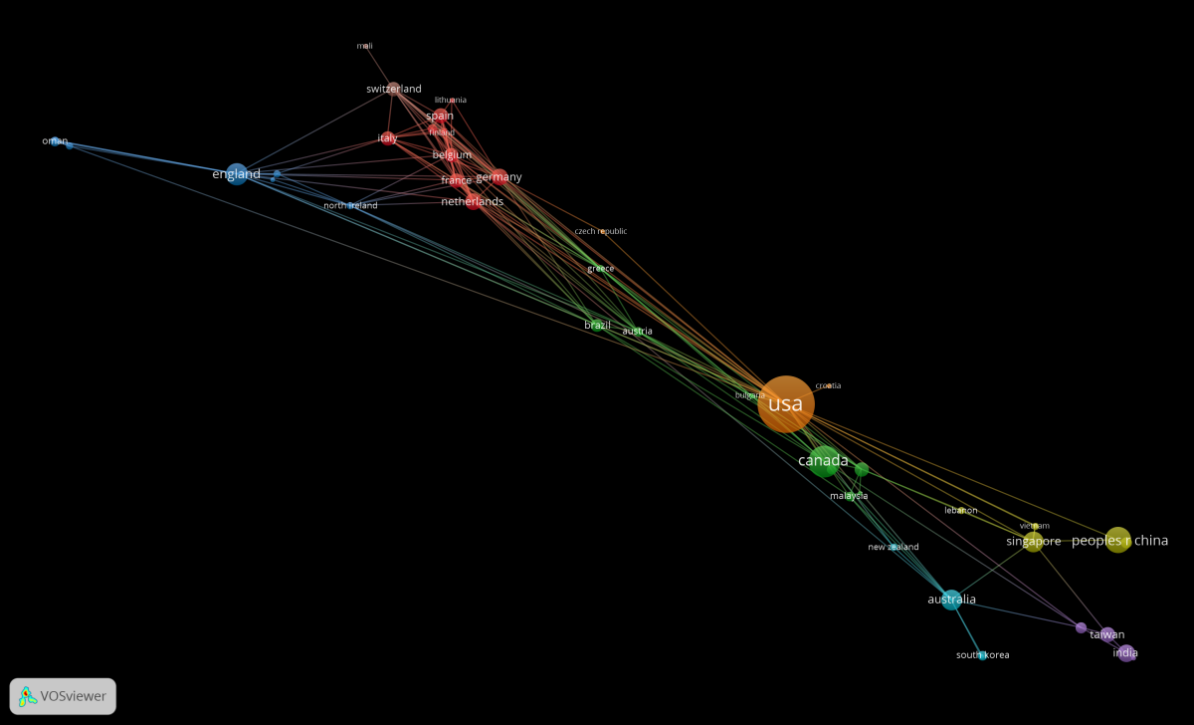
National and regional co-occurrence map.

Based on a comprehensive national analysis, 57 countries globally contributed to the exploration of AI within the field of medical education from 2013 to 2022. The United States took the lead by publishing 66 papers, thereby establishing itself as the most actively engaged country in this domain. The subsequent countries, albeit with lesser contributions, were Canada (24 papers), China (17 papers), England (13 papers), Singapore (12 papers), Australia (12 papers), India (9 papers), Germany (8 papers), the Netherlands (8 papers), and Spain (7 papers). The most cited countries were the United States (845 citations), Singapore (489 citations), and China (435 citations). When evaluated in terms of total link strength, the United States (44), the Netherlands (29), and Belgium (26) emerged as the top 3 countries (Table 2). Figure 2 shows that a clear inclination of North American and European countries toward the application of AI in medical education is evident, possibly due to their technological advancement. The United States has been a front-runner in this arena, publishing a multitude of relevant papers. Concurrently, it has fostered collaborative relationships with various countries for related research.

**Table 2.** Top 10 publications, centrality, and citations of countries.

Rank	Documents	Countries	Citations	Countries	Total link strength	Countries
1	66	United States	845	United States	44	United States
2	24	Canada	489	Singapore	29	The Netherlands
3	17	People’s Republic of China	435	People’s Republic of China	26	Belgium
4	13	England	371	Canada	23	Germany
5	12	Australia	155	England	22	England
6	12	Singapore	108	Spain	20	France
7	9	India	101	Germany	19	Italy
8	8	Germany	94	The Netherlands	19	Switzerland
9	8	The Netherlands	94	Belgium	18	Spain
10	7	Spain	85	Iran	16	Greece

### Institutional Analysis

**Figure 3.**
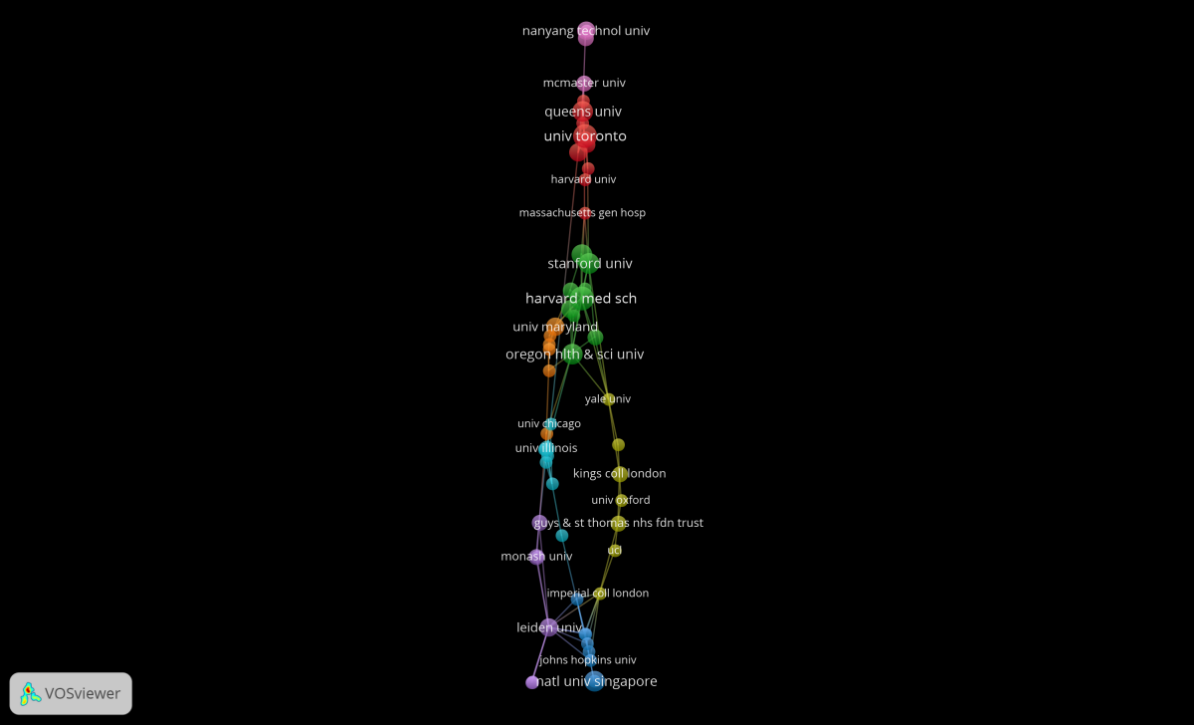
Organizations co-occurrence map.

Shifting the focus to an institutional analysis reveals that from 2013 to 2022, 77 institutions were engaged in research on AI in medical education. The two institutions that topped the list in terms of the number of publications were Harvard Medical School and the University of Toronto, each with 7 contributions, followed by McGill University and the National University of California, San Francisco (5 contributions each) (Table 3). The institutions receiving the most citations were Nanyang Technological University (396 citations), McGill University (149 citations), and the University of Chicago (127 citations). Figure 3 shows that Leiden University and Harvard Medical School demonstrated more collaboration with other institutions, both exhibiting a link strength of 15.

**Table 3.** Top 10 publications, centrality, and citations of organizations.

Rank	Documents	Organization	Citations	Organization	Total link strength	Organization
1	7	Harvard Medical School	396	Nanyang Technological University	15	Leiden University
2	7	University of Toronto	149	McGill University	15	Harvard Medical School
3	5	McGill University	127	University of Chicago	11	Oregon Health and Science University
4	5	National University Singapore	104	University of British Columbia	10	University of Toronto
5	5	Oregon Health and Science University	86	Guy’s and St Thomas’ NHS Foundation Trust	9	University of British Columbia
6	5	Queens University	83	Kings College London	9	Stanford University
7	5	Stanford University	68	University California San Francisco	9	Queens University
8	5	University of California San Francisco	67	National University Singapore	8	Imperial College London
9	4	Emory University	66	Sultan Qaboos University	8	Johns Hopkins University
10	4	Leiden University	60	University of Maryland	7	Ludwig Maximilians University Munchen

### Author Analysis

**Figure 4.**
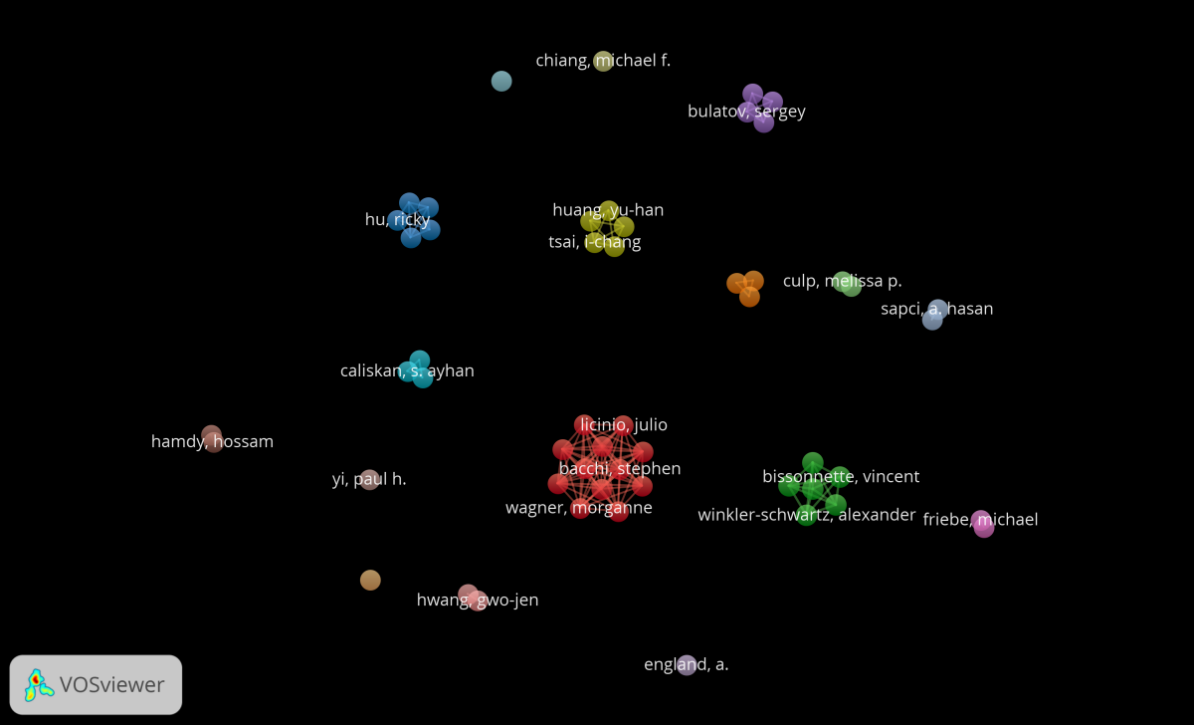
Authors’ co-occurrence map.

In the span of the last decade, research on AI and medical education has seen the involvement of a total of 53 authors. The authors most frequently contributing to the documents included Vincent Bissonnette, Charlotte Blacketer, Rolando F Del Maestro, Nicole Ledwos, Nykan Mirchi, Alexander Winkler-Schwartz, and Recai Yilmaz, each writing 3 papers. The authors garnering the highest citations encompassed the same group, with each achieving 143 citations (Table 4). As discerned from the VOSviewer image, there are no researchers with a significantly high number of publications, indicating that the volume of published papers remains relatively minimal. Figure 4 shows that research in this field is still nascent, with no particular research team outperforming others.

**Table 4.** Top 10 publications, centrality, and citations of authors.

Rank	Documents	Author	Citations	Author	Total link strength	Author
1	3	Bissonnette, Vincent	143	Bissonnette, Vincent	22	Bacchi, Stephen
2	3	Blacketer, Charlotte	143	Del Maestro, Rolando F	22	Duggan, Paul
3	3	Del Maestro, Rolando F	143	Ledwos, Nicole	22	Gallagher, Steve
4	3	Ledwos, Nicole	143	Mirchi, Nykan	22	Licinio, Julio
5	3	Mirchi, Nykan	143	Winkler-Schwartz, Alexander	22	Parnis, Roger
6	3	Winkler-Schwartz, Alexander	143	Yilmaz, Recai	22	Perry, Seth W
7	3	Yilmaz, Recai	56	Culp, Melissa P	22	Symonds, Ian
8	2	Bacchi, Stephen	56	Mollura, Daniel J	22	Tan, Yiran
9	2	Bulatov, Sergey	47	Sapci, A Hasan	22	Thomas, Josephine
10	2	Caliskan, S Ayhan	47	Sapci, H Aylin	22	Wagner, Morganne

### References Analysis

In accordance with Table 5, there are 15 papers that serve as primary references in the research of AI and medical education. The paper titled “Medical Students’ Attitude Towards Artificial Intelligence: A Multicenter Survey” emerged as the most frequently cited and most pertinent literature, garnering 36 and 109 citations, respectively. It primarily evaluates the attitudes of undergraduate medical students toward radiology and medical AI.

**Table 5.** Top 10 publications, centrality, and citations of cited reference.

Rank	Citations	Cited reference, year	Total link strength	Cited reference, year
1	36	Dos Santos et al [14], 2019	109	Dos Santos et al [14], 2019
2	23	Kolachalama and Garg [15], 2018	103	Wartman and Combs [16], 2018
3	23	Sit et al [17], 2020	98	Kolachalama and Garg, 2018 [15]
4	21	Gong et al [18], 2019	96	Sit et al [17], 2019
5	21	Wartman and Combs [16], 2018	85	Masters [19], 2019
6	19	Paranjape K et al [20], 2019	81	Paranjape K et al [20], 2019
7	19	Topol [21], 2019	78	Topol [21], 2019
8	16	Chan and Zary [8], 2019	78	Wartman and Combs [22], 2019
9	16	Masters [19], 2019	78	McCoy et al [23], 2020
10	15	Wartman and Combs [22], 2019	75	Park et al [24], 2019

**Figure 5.**
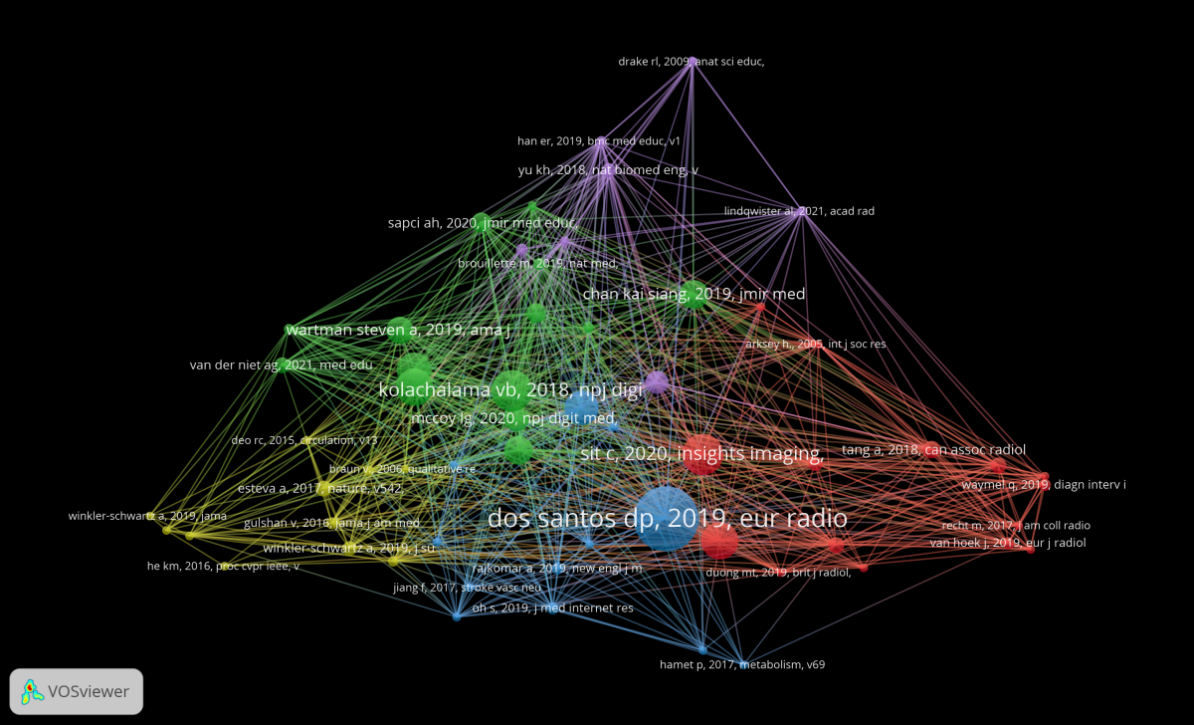
Cited reference co-occurrence map.

The papers “Machine Learning and Medical Education” and “Attitudes and Perceptions of UK Medical Students Towards Artificial Intelligence and Radiology: A Multicenter Survey” are the second most frequently cited. The papers “Medical Education Must Move From the Information Age to the Age of Artificial Intelligence” and “Machine Learning and Medical Education” occupy the second position in terms of total link strength. Figure 5 illustrates this information.

### Keywords Analysis

**Figure 6.**
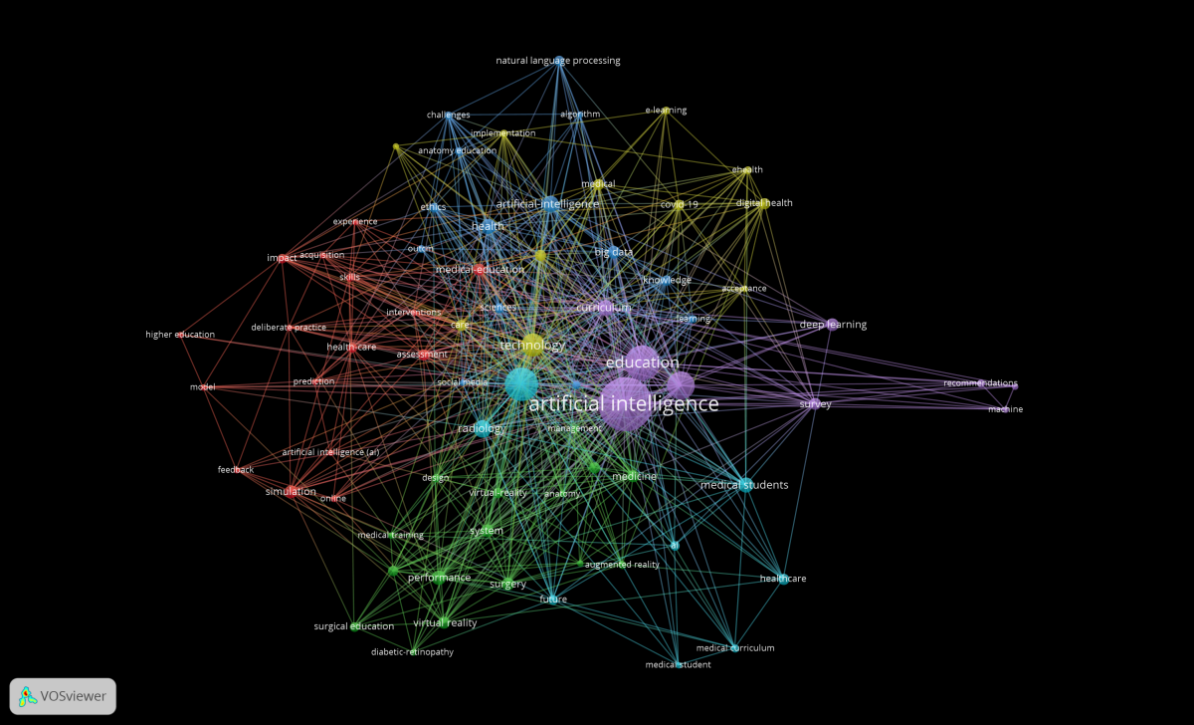
Keywords co-occurrence map.

The study examining AI and medical education from 2013 to 2022 concentrated on 39 primary keywords (Table 6). Figure 6 shows that AI (100), education (47), and medical education (45) have the highest frequency and connection intensity.

**Table 6.** Top 10 keywords related to AI in medical education.

Rank	Occurrence (%)	Keywords	Total link strength	Keywords
1	100	AI^a^	259	AI^a^
2	47	Education	131	Education
3	45	Medical education	114	Medical education
4	33	Machine learning	107	Machine learning
5	23	Technology	94	Technology
6	15	Radiology	56	Curriculum
7	14	Artificial intelligence	43	Radiology
8	13	Curriculum	43	Artificial-intelligence
9	12	Health	41	Performance
10	12	Medical students	38	Health

a AI: artificial intelligence.

### Research Status

**Figure 7.**
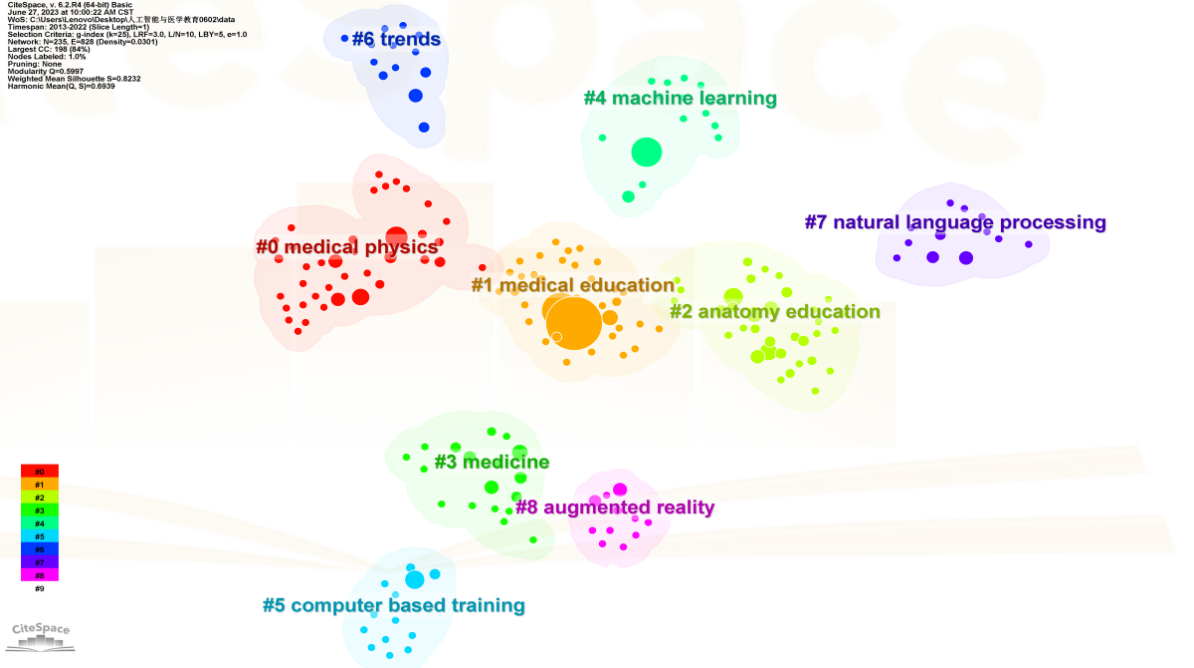
Research status map.

Figure 7 shows that the analysis of references with high citation frequency and centrality enables us to understand highly respected research results in the application of AI in medical education.

In clusters 0 and 1, the swift advancement of AI has led to its application across all medical sectors, notably radiology [25-27]. Despite radiologists, residents, and medical students increasingly recognizing the importance of understanding AI, medical education that targets future radiologists is only just commencing [14,20,28]. Current investigations fall into 3 categories, that are (1) methods to facilitate medical students in learning AI knowledge, (2) using AI technology to augment radiology teaching efficiency and assist medical students in identifying clinical images, and (3) medical students’ attitudes toward AI application in radiology. An AI curriculum (Artificial Intelligence in Radiology [AI-RADS]) has been devised to equip residents devoid of computing backgrounds with basic AI knowledge and its radiology application. The curriculum was highly rated (9.8 out of 10) by residents for overall satisfaction and significantly increased students’ confidence in interpreting AI-related journal papers. There was a marked improvement in residents’ comprehension of AI’s fundamental concepts [29]. Some institutions emphasize integrating AI frameworks to strengthen radiology education. For example, after scanning, the patient's condition will be interpreted by artificial intelligence to give a preliminary diagnosis. AI assigns cases to interns whose personal profiles indicate that they will benefit the most. Interns cooperate with artificial intelligence and use equivalent tools for diagnosis. Interns and attending radiologists elaborate on the final report. AI uses natural language processing to anonymize new cases, add them to the teaching archive, and update the personal profiles of trainees after new cases are completed. When trainees review cases similar to new cases, AI will provide them with corresponding cases from the teaching archive.[30]. As this framework continues to evolve, it may be possible to achieve “precise medical education” tailored to the individual learning styles and needs of the students [30]. A multicenter survey assessing UK medical students’ attitudes and perceptions of AI and radiology revealed that students recognize the significance of AI and are eager to engage [17]. This prompts the need to integrate relevant AI courses into medical education to acquaint students with practical AI applications and constraints, thereby maintaining their learning enthusiasm and preventing AI-related panic.

Natural language processing is an important direction in the fields of computer science and AI. It studies various theories and methods that enable effective communication between humans and computers using natural language. Its main function here is to distinguish rare cases

In cluster 2, eHealth refers to the use of information and communication technologies to fulfill health care needs in various domains, including AI, telemedicine, Internet of Things, connected devices, and mobile health (mHealth) [31]. eHealth technologies provide access to health care in rural areas and support the management of numerous health conditions [32-36]. Following the release of the World Health Organization’s national eHealth strategy tool in 2012, it is imperative for future medical students to receive eHealth education and training. Current medical education primarily includes conceptual courses while neglecting practical training [37]. While emphasizing the inclusion of eHealth in medical education, it is also important to recognize the potential adverse outcomes of over-reliance on AI technology [38]. Hence, identifying the optimal eHealth application areas in health care is necessary [39].

In cluster 3, the integration of medical education and AI holds significant value and potential beyond radiology, extending into surgical education and surgery. AI’s earliest medical applications were in image-based specialties, such as radiology, pathology, ophthalmology, and dermatology. However, its application in procedural professions such as surgery may require more time [40,41]. The benefits of AI application in surgery mainly include integrating preoperative, intraoperative, and postoperative data to improve the accuracy of the clinical decision-making system and predict postoperative complications more efficiently and applying surgical knowledge and education to interact with surgeons and patients through virtual or augmented reality. For instance, virtual reality simulators were initially used in laparoscopic surgery training [42]. A study involving 176 medical students was conducted to assess the accuracy of robot-assisted virtual surgical simulations after integrated deep learning, showing improved accuracy [43]. In 2022 and 2023, AI application breakthroughs were achieved in oral and maxillofacial surgery education [44] and orthopedic surgery [45]. While AI proves beneficial in surgery and surgical education, especially in surgical ability assessment, it raises questions regarding whether AI can ever match the intelligence and audacity of the human educators. Although advanced AI teaching tools can be incorporated into surgical education, current technology cannot fully replace multifaceted surgeons or surgical educators. Addressing the transparency and responsibility of AI application in medical education and resolving ethical issues may require more time and effort.

In cluster 5, the rapid AI development profoundly impacts medical education. Modern medical education must accommodate various health care systems, including digital health systems and big data generation in a highly connected world [46]. A Canadian survey of medical students’ perceptions of AI’s impact on radiology in 2018 showed that anxiety induced by the prospect of AI replacing radiologists deterred many students from considering radiology [18]. The radiology community should appreciate AI’s potential impact on the profession, educate students appropriately about AI’s role, and ensure radiology’s viability as a long-term career option. While AI’s benefits in medicine include eliminating human bias and enhancing pattern recognition and decision-making, its drawbacks, such as the inability to provide warmth and empathy to patients and absorb the wisdom of human educators, should not be underestimated. The confusion about whether AI’s role in medical education is supplementary or replacement-based is another concern [47]. In summary, while AI promises great advances and changes in medicine, it also poses numerous challenges and problems. The medical community needs to proactively address these challenges, leverage AI technology benefits, and promote continuous innovation and improvement in medical services.

### Research Frontier

**Figure 8.**
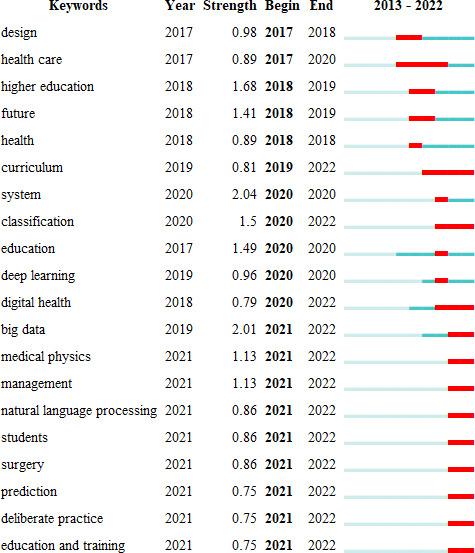
Top 20 keywords with strongest citation bursts.

Figure 8 shows that big data has a significant intensity of 2.01, firmly at the top of the list, and has become the focus of medical education in the past 3 years. The emergence and proliferation of COVID-19 in 2019 ushered in the big data epoch in medicine, with telemedicine systems, clinical intelligent decision-making, and management systems taking on pivotal roles.

First, the advent of big data has catalyzed the innovation of medical teaching paradigms: what does the future hold for medical education in the digital age? A study conducted by Han et al zeroes in on a future medical education model that leans heavily on big data, cutting-edge technology, and AI, with the aim to cultivate a new breed of medical students who display enhanced humanistic attributes, co-operation capacity, patient-needs sensitivity, and societal and global orientation [46].

Second, big data has stimulated innovation in clinical medicine models: the integration of advanced technologies like machine learning, clinical intelligent decision and management systems, and electronic medical records has propelled shifts, innovation, and advancement within clinical medicine paradigms. The study by Kolachalama and Garg posits that AI, fueled by machine learning algorithms, is an emerging computer science branch that is swiftly gaining traction in health care. AI is anticipated to play an instrumental role in precision medicine and health [15]. In 2022, Watson and Wilkinson released a paper entitled “Digital Healthcare in COPD Management: A Narrative Review on the Advantages, Pitfalls, and Need for Further Research,” illustrating the vast potential of digital health care innovation [48]. During the COVID-19 pandemic, it was expected that big data would mitigate the workload for doctors interpreting digital data, enhance their diagnostic and prognostic abilities, equip clinicians with intelligent decision-making and management systems, and offer patients optimal clinical care and self-management strategies.

Undeniably, big data, akin to many emergent tools, is a double-edged sword. Ensuring its tailored use and dialectical treatment constitutes a crucial aspect of digital health, striving to exploit its merits while circumventing its demerits. The pursuit of enduring, comprehensive, and precise population health data management emerges as a long-term strategy.

The recent surge in terms indicates that “management” is intimately linked to “big data.” Confronting the colossal medical data of today, the incorporation of AI technology can enhance management efficiency in spheres, such as hospital medical management, disease surgery management, and chronic disease management, among others. AI algorithms are used to scrutinize data pertaining to patients’ hospitalization duration, hospitalization route, and climatic and temporal factors, which effectively curtail the hospitalization duration and significantly rectify issues, such as the misallocation of medical resources [49]. Leveraging a diabetic retinopathy automatic grading and training system furnished with an AI-driven diagnosis algorithm to groom budding doctors can augment diagnostic accuracy, thereby strengthening DR management [50]. Surgical video, a crucial data source for medical education, should be systematically stored and managed. A system intended to assist doctors in managing surgical videos can heighten the efficiency of continuing education by dissecting surgical videos and marking critical segments or frames to generate AI reports [51].

## Discussion

In this investigation, a bibliometric evaluation of 195 pertinent papers over the preceding decade was meticulously executed using CiteSpace and VOSviewer. This research illustrates the findings related to countries, institutions, authors, citations, and keywords using tables and diagrams, offering an analytical perspective on the current research status and emerging frontiers in this domain. The outcomes were exhaustively analyzed.

Initially, examining the annual publication count, authors, institutions, and countries, it was identified that from 2019 onwards, global interest and recognition of AI’s applicability in medical education experienced an upswing. Second, superficially, collaboration in this arena might appear limited, an aspect that can be attributed to this field’s unique nature and the diverse modalities of medical education across different regions. For future progress, it is recommended that countries focus on harmonizing their approaches while acknowledging their differences, fostering collective advancement, and advocating for a mutual elevation of medical education standards.

Furthermore, an evaluation of the current research status and prevalent research themes highlighted that the extent of AI technology integration in medical education is significantly inadequate, with a rather limited focus area. Consequently, it is advocated that future efforts should aim at active exploration to unearth novel advancements.

Finally, AI, being inherently enigmatic, evokes uncertainty among both educators and learners about its future potentialities. Therefore, the immediate concern should be to strategically leverage its potential while mitigating its drawbacks, which, indeed, becomes the highest priority for future advancement.

Some limitations should be considered. The search strategies used can potentially yield divergent results, and the strategy opted for in this study might not encompass all pertinent literature. With the swift advancement of AI, several papers in this domain were brought to light in 2023. However, the temporal span of this study extends from 2013 to 2022, thus excluding the contributions from 2023.

The study highlights the promising potential of AI in medical education research, emphasizing the need for enhanced interregional collaboration and improved research quality. These insights provide valuable guidance for future research directions.

## Abbreviations

AI: artificial intelligence
AI-RADS: Artificial Intelligence in Radiology
mHealth: mobile health
